# Safety of Duodenal Endoscopic Submucosal Dissection for Superficial Non-Ampullary Duodenal Epithelial Tumor: A Single-Center Study in the United States

**DOI:** 10.3390/jcm13010143

**Published:** 2023-12-27

**Authors:** Mako Koseki, Makoto Nishimura, Tarek Nammour, Kana Chin, Sayaka Nagao, Jacques C. Beauvais, Mark A. Schattner

**Affiliations:** 1 Gastroenterology, Hepatology, and Nutrition Service, Memorial Slone Kettering Cancer Center, New York, NY 10022, USAschattnm@mskcc.org (M.A.S.); 2 Internal Medicine, Mount Sinai Beth Israel, New York, NY 10003, USA

**Keywords:** endoscopic submucosal dissection, superficial non-ampullary duodenal tumors, en bloc resection

## Abstract

Endoscopic submucosal dissection (ESD) of superficial non—ampullary duodenal epithelial tumors (SNADETs) is associated with a high rate of en bloc resection and low rate of recurrence. However, in the United States, SNADETs are predominantly managed using endoscopic mucosal resection (EMR) or surgery because the feasibility and safety of duodenal ESD have not yet been established. In this study, we analyzed the outcomes of duodenal ESD for SNADETs. This single—center retrospective study reviewed the data of patients who underwent ESD for SNADETs between June 2018 and August 2023. Baseline patient characteristics, histopathology of the resected lesions, adverse events, and recurrence rates were evaluated. The primary outcome measures were en bloc resection, complications, and recurrence rate. Thirty ESD procedures were performed on 24 patients. All 30 lesions were adenomas, with no cancerous lesions. The en bloc resection rate and R0 resection rates were both 53%. There were no cases of procedure-associated perforation. Post-ESD bleeding was observed in six cases. No ESD—related mortality was observed. The recurrence rate was 14% in 1 year follow up, and 28% the during all follow-up period. ESD is a safe option for SNADET in the United States; however further comparative studies are necessary to determine the optimal procedure for North American populations.

## 1. Introduction

Endoscopic submucosal dissection (ESD) is a well—established and effective endoscopic technique that facilitates removal of epithelial lesions throughout the gastrointestinal tract. However, performing duodenal ESD remains challenging because of the high risk of severe complications due to the thin duodenal wall and unstable scope maneuverability. Duodenal ESD is reported to be effective for achieving en bloc resection and preventing local recurrence in Eastern Asian countries, yet it remains a rarely performed procedure in the United States [1]. To date, most duodenal tumors in the U.S. have been removed with either with endoscopic mucosal resection (EMR) or surgery because the feasibility and safety of duodenal ESD have not been established.

Superficial non-ampullary duodenal epithelial tumors (SNADETs) are duodenal adenomas with malignant potential, including adenocarcinomas that originate above the mucosal layer. SNADETs occur sporadically or in association with familial syndromes such as familial adenomatous polyposis (FAP) and Lynch syndrome [2]. Approximately 40% of duodenal adenomas are sporadic, whereas the remaining 60% are present in patients with familial syndromes. Sporadic duodenal polyps are uncommon in Western countries and are detected in up to 5% of the patients referred for upper gastrointestinal endoscopy. In contrast, many individuals in Western countries have hereditary gastrointestinal polyposis and could benefit from the complete removal of these polyps in the duodenum.

Duodenal adenocarcinoma is a rare, but aggressive form of malignancy [3]. SNADETs represent early-stage adenocarcinoma or benign precancerous lesion with the potential to progress to adenocarcinoma [4]. Studies have indicated that in more than 30% of cases in which initial biopsies do not indicate cancer, post-resection pathological examination shows an upgrade from high- or low-grade adenoma to adenocarcinoma [5]. For sporadic adenomas, the risk of malignant transformation is approximately 30–50% [6,7,8]. For duodenal adenomas in hereditary gastrointestinal polyposis syndromes, the risk is estimated to be 7–36% over 10 years [9]. Therefore, it is recommended that adenomas in the duodenum be resected in all cases, regardless of the size and biopsy results. Currently, there are no guidelines in the U.S. addressing this however, the European Society of Gastrointestinal Endoscopy (ESGE) recommends that all duodenal adenomas should be considered for endoscopic resection because progression to invasive carcinoma is highly likely [10].

Herein, we aimed to analyze the outcomes of duodenal ESD and demonstrate its safety of ESD for duodenal neoplasms in the U.S.

## 2. Materials and Methods

### 2.1. Study Population

This single—center retrospective study was approved by the Institutional Review Board of the Memorial Sloan Kettering Cancer Center (20-495 A) [2], and informed consent was obtained from all patients for the ESD procedures. We retrospectively reviewed the data of all the patients who underwent ESD for SNADETs between June 2018 and August 2023 at our center. The eligibility criteria for ESD were as follows: (i) non—ampullary tubular adenoma diagnosed using biopsy or previous interventions, (ii) tumors limited to the mucosa, and (iii) lesions considered difficult to resect with EMR, including those in which EMR failed or was aborted due to the size, shape, and location of the lesion. Some of these cases were referred to our institute from other facilities after failed EMR or polypectomy, and some were from other endoscopists who had decided that the lesions were not an indication for EMR.

A total of 35 ESD procedures for duodenal tumors were performed during the study period. Five cases were excluded from this study because the pathological results showed neuroendocrine tumors in three cases, ampullary adenoma in one case, and normal mucosa in one case. Therefore, 30 SNADETs in 24 patients were evaluated in this study.

### 2.2. Evaluation of Baseline Patient Characteristics

We assessed the patients’ baseline demographic and clinical characteristics, including age, sex, ethnicity, history of any type of cancer, history of hereditary gastrointestinal polyposis syndromes, use of anticoagulation and antiplatelet medications, and history of prior duodenal interventions. Additionally, the American Society of Anesthesiologists scores were reviewed.

### 2.3. Patient Procedural Course

Patients were instructed to stop consuming food orally at midnight on the day of the procedure. Anticoagulation medications were administered accordingly to the American Society for Gastrointestinal Endoscopy (ASGE) guideline. Patient presented to our facility on the day of the procedure. After the procedure, the patients were admitted for one or two nights. If no signs of complications were observed after the procedure, the patients were able to start eating a clear liquid diet on the same day. The following day, after confirming stable blood tests, the diets were advanced to a full liquid diet, and if they tolerated the food well, they were discharged.

### 2.4. Endoscopic Submucosal Dissection Procedure

All ESD procedures were performed under general anesthesia using endotracheal intubation in endoscopy units. ESD was performed using a standard upper endoscope (GIF-H 190; Olympus America, Center Valley, PA, USA) in all the cases. Submucosal injections were performed using ORISE gel (Boston Scientific, Marlborough, MA, USA; no longer available due to recall), Eleview (Medtronic, Louisville, CO, USA), Blue Eye (Omnimed, Winchester, UK), or EndoClot SIS (Olympus America, Center Valley, PA, USA). For cutting, a needle-type knife, a 1.5 mm or 2.0 mm ORISE ProKnife (Boston Scientific, Marlborough, MA, USA) or DualKnife J (Olympus America, Center Valley, PA, USA), was used. First, mucosal markings were made at 2 mm intervals around the lesion using a needle-type knife. Subsequently, submucosal injection was performed to elevate the lesion. Mucosal incisions were made using a needle—type knife and the submucosal layers were dissected using a dedicated knife for complete removal of the lesion. ProKnife was used in 13 cases and DualKnife J in 17 cases; however, the protocol for the ESD remained consistent across cases. If necessary, submucosal injection was repeated during the procedure with an injection needle or through a knife, and carbon dioxide was used for insufflation. Endoscopic hemostasis was achieved using a Coagrapser (Olympus America, Center Valley, PA, USA). For cases with mild fibrosis or without prior intervention or scarring of the lesion, the standard ESD method, as described above, was used. In cases in which fibrosis was severe, the pocket creation method or underwater method was performed, depending on how challenging the lesions were. No tracking technique was necessary to perform safe ESD for in all cases. In some cases, additional snaring was performed for possible leftover of the lesions.

After specimen removal, closure using adequate endoscopic clips using with Resolution ^TM^ (Boston Scientific, Marlborough, MA, USA) was performed in most cases. No special suture technique was used, and the closure was performed at the discretion of the endoscopist. Specimens were extracted orally and placed in formalin.

All the ESD procedures were performed by an experienced endoscopist (M.N.). The endoscopist has performed approximately 1700 cases of ESD, as well as 20 peroral endoscopic myotomy procedures, and has 21 years of experience with advanced endoscopy. Our facility performs approximately 150–200 ESD procedures per year in one endoscopic unit, which is mainly performed by the endoscopist.

### 2.5. Evaluation of Duodenal ESD

We reviewed the size, location, and duration of the dissection for each lesion. The area of the resected specimen (mm^2^) was calculated using the following formula: major axis (mm)/^2^ × minor axis (mm)/2 × 3.14 [11]. The procedure time (min) was defined as the time from the start of marking to the completion of the resection. Dissection speed (mm^2^/min) was calculated as the area of the resected specimen (mm^2^)/dissection time (min).

### 2.6. Histological Assessment

For pathological evaluation, all resected specimens were fixed in formalin and sectioned to assess tumor involvement. Subsequently, the presence of malignant lesions or high—grade adenomas and lateral and deep margins were assessed. All the resected specimens were evaluated by an experienced pathologist specializing in gastroenterology. Unlike some Eastern Asian countries where specific guidelines are available for histologic classification, there are no guidelines in the U.S. for histologic classification of neoplasms removed specifically by endoscopic procedures. Therefore, the pathologists used the WHO Classification of Tumors for the pathological evaluation.

En bloc resection was defined as tumor removal in a single piece. R0 resection was defined as complete resection with histologically confirmed tumor—free margins.

### 2.7. Adverse Events

Post—ESD bleeding was defined as bleeding that occurred after ESD and required additional endoscopy. Perforation was defined as the creation of a hole connecting the duodenal tract to peritoneal cavity.

### 2.8. Follow-Up

Post-treatment surveillance for recurrence was performed for in all patients. Endoscopic examination and evaluation were recommended to be performed 6–12 months after ESD, depending on the lesion size, pathological results, and patient preference. In cases of positive margins, patients were offered repeat endoscopic procedures, such as ESD, EMR, or argon plasma coagulation (APC), and were closely monitored for any sign of recurrence. The prognosis of patients whose follow—up period was more than 12 months was analyzed for follow-up recurrence.

## 3. Results

### 3.1. Patient Characteristics

During the study period, 30 ESD procedures were successfully performed on 24 patients. The median age was 68 years (range, 47–77 years), comprising 14 male and 16 female cases. (24 patients: 12 male and 12 female). Regarding ethnicity, 26 patients were white, and 4 were African Americans. No other ethnicity was present in our study. Of the 30 cases, four had FAP, two had MUTYH-associated polyposis (MAP; another type of polyposis-related hereditary condition similar to FAP), and two had Lynch syndrome. Approximately half of the patients had a history of cancer involving any organ prior to intervention. The resected lesions were located in the second, third, and fourth portions of the duodenum in 70% (21/30), 27% (8/30), and 3% (1/30) of the cases, respectively. A history of interventions, including EMR, polypectomy, and ESD, was observed in 10 patients. Some histories may have been missed, as many records of patients who were referred from other facilities could not be obtained with intact information. The baseline characteristics of the study population are presented in Table 1.

### 3.2. Endoscopic Procedure

The median procedure time was 142 min, with an interquartile range (IQR) of (100–183) (N = 24). The median size of the dissected lesion was 30.5 (18–40) mm (N = 30). The median dissection speed for cases in which both area and time were available was 4.27 (3.24–5.42) mm^2^/min (N = 23). A median volume of 38 mL (30–70 mL) of the injection liquid was used for the procedures (N = 30). The lesion was closed using endoscopic clips in 27 of the 30 patients. The details of the endoscopic procedures are shown in Table 2. When it was challenging to reach the third or fourth portion of the duodenum using upper endoscopes, the stomach was deflated, and the reach was attempted again. The underwater technique was used in some cases to reach the lesion. All lesions could be reached using standard upper endoscopes, without running out of the scope.

### 3.3. Histology

Thirty lesions were evaluated by the pathologists at our facility. All 30 lesions were identified as adenomas with no cancerous lesions in the tumor. Of these, four adenomas included portions of high-grade tumors. The en bloc and R0 resection rate were 53% (16/30), respectively. The lateral margin was positive in six lesions, and eight lesions could not be assessed due to fragmentation. The deep margin was positive in four lesions, and eight lesions could not be assessed because of fragmentation.

### 3.4. Complications

The median hospitalization duration was 2 days, ranging from 0 to 7 days. No perforations occurred during or after the procedures. Post—ESD bleeding was observed in six cases: four during hospitalization and two post—discharge. Bleeding during hospitalization necessitated repeat endoscopy in all four patients. Two lesions required coagulation and clipping, and two lesions required no intervention, as the bleeding had stopped with no signs of residual bleeding. Regarding the bleeding that occurred after the patients were discharged, one patient returned to the hospital 4 days after ESD and underwent endoscopic examination with clipping for hemostasis. Another patient returned 13 days after ESD and underwent endoscopy, without any signs of bleeding. Regarding delayed complications, two patients developed duodenal stenosis after ESD, both of whom underwent multiple dilation procedures. Among these two cases, one patient had 4/5 circumferential resections and the other had 2/3 circumferential resections. There were no deaths related to ESD.

### 3.5. Follow-Up 

Among the 30 cases, there were 21 cases in which the follow—up period was more than 12 months. Nine cases were not followed up, as five cases were performed within 12 months of the analysis date, and four cases either did not return or declined to undergo endoscopic evaluation after the procedure. Of the 21 cases, three experienced recurrences at the same site at the 1 year follow-up (14%).

The follow-up period beyond 1 year differed for each case depending on the histological results and endoscopic findings. The median follow—up period of the 21 patients was 29 months (IQR (19–48)). Three more cases of recurrence at the ESD lesion were observed at 14, 21, and 25 months each. In total, six patients experienced recurrences at the initial ESD site during the follow-up period (28%). Among the six cases, one patient had FAP. 

For the treatment of local recurrences, all six cases underwent repeat endoscopic procedures for the same lesions. For five cases, ESD procedures were repeated. On repeat ESDs, three cases were R0 resection; however, recurrence was observed within 2 years in one case, which prompted another ESD procedure. In two cases, the lesions were fragmented during resection; of them, one required another ESD, and one did not experience further recurrence. For one case in which ESD was not repeated for six recurrences, hybrid EMR was performed, which successfully led to R0 resection. As of now, none of the six cases shown any recurrence. 

Of note, during the follow—up period, another duodenal tumor at a different location was observed in five cases, for which either hybrid EMR or ESD was performed. Of these five cases, three were on patients with FAP and one was on MAP. One patient had no history of hereditary polyposis.

## 4. Discussion

Research on endoscopic interventions for SNADETs in North America is limited. Our study demonstrated that ESD is a safe method for resecting SNADETs in the U.S. While the standardization of treatment for SNADETs is still developing, establishing a standardized endoscopic approach to avoid pancreaticoduodenectomy for these tumors would be beneficial for patients. Over the past decade, ESD has emerged as a viable treatment option for SNADETs in East Asian, and treatment outcomes have been documented extensively. However, data regarding the outcomes in the North American population are still lacking. Notably, the only meta—analysis summarizing EMR and ESD outcomes for SNADETs included studies primarily from Asian countries, Belgium, and the United Kingdom, did not include the North American population [12]. Our findings indicate that duodenal ESD is a viable treatment modality for SNADETs in North American setting.

EMR is generally safer than ESD and is the preferred method for resecting SNADETs in endoscopic procedures worldwide. Furthermore, underwater EMR (UMER) has been shown to have high efficacy and a high rate of en bloc resection for SNADETs [13], with various techniques developed to improve UMER success rates [14]. However, for lesions larger than 20 mm, the possibility of EMR achieving en bloc resection is limited because of the size constraints of mucosa that snares can encapsulate [15]. Therefore, ESD is recommended for large lesions. In addition, in cases where patients have multiple previous EMRs or tattoos injections in the same area, fibrotic tissue can make EMR challenging or impossible due to non-lifting. In such cases, ESD tends to yield better outcomes, even for smaller lesions.

Generally, the incidence of complications during and after endoscopic interventions for SNADETs is significantly higher than that for other segments of the digestive tract [16]. Adverse events are a major concern when performing ESD for SNADETs, contributing to the lack of standardized endoscopic procedures. This could be attributed to anatomical features such as a narrow lumen, abrupt flexures leading to poor endoscopic stability, presence of Brunner’s glands in the submucosal layer causing rigidity in the duodenal wall, and a thin muscle layer associated with an elevated risk of perforation [17]. In addition, exposure of the resected lesion to bile and pancreatic juices from the duodenal papilla elevates the risk of bleeding and perforation, especially in delayed events [16]. Previous reports from Asian countries on the outcomes of endoscopic treatment for SNADETs have shown a perforation rate is of 13–50% and a posterior bleeding rate of approximately 20% [18]. A study conducted in the U.S. reported that the overall bleeding rate after EMR was 16% [19]. In our study, we noted an overall bleeding rate of 20% (6/30 cases), which was similar to previous data from other countries. Notably, no perforation was recorded during or after the procedure.

Our patient cohort was unique compared with that of previous studies. As stated, polypectomy and EMR are more commonly performed in U.S. hospitals for SNADETs. Our facility is in charge of cases in which the lesion was either too large or flat for EMR and polypectomy, or multiple failed attempts of prior interventions necessitated complete resections in an alternative way. Many failed cases had tattoos and scars. Even in these challenging cases, no perforations were observed in any patients. The endoscopist took measures to avoid damage to the muscularis propria on all occasions, as precise contact with fibrotic lesions were more challenging. Additionally, unlike ESD in other organs, the endoscopist chose not to use any traction device and decided to use the standard endoscopes instead of longer endoscopes such as PCF to maintain better maneuverability. Such skilled performance of the procedure led to the occurrence of no perforation.

SNADETs have high recurrence rates. Some studies have indicated that ESD may lead to a lower recurrence rate than EMR [12]. One study documented a 15% recurrence rate over a median follow-up period of 6–72 months after EMR, while other studies reported recurrence rates ranging from 0–37% [5]. In our study, six out of the 21 cases that were followed for a median follow—up period of 29 months experienced recurrences at the same site resulting in a recurrence rate of 28%. With appropriate treatments for these recurrent cases, all six cases are now completely treated, with no recurrence. Although our data are limited, this suggests that the recurrence rate may be comparable to that of EMR.

Furthermore, in our cohort, 47% of the patients had a history of cancer of any organ, 20% had FAP or MAP, and 7% had Lynch syndrome. One of the six patients who experienced recurrence had hereditary polyposis conditions, and five had sporadic conditions. This implies that lesions secondary to hereditary polyposis do not specifically have indicate high recurrence rates. Conversely, as hypothesized, patients with FAP or MAP are more likely to undergo procedures in different sites of the duodenum, requiring further interventions. In contrast, no such correlation was observed in patients with Lynch syndrome or history of cancer.

Duodenal ESD is advantageous for achieving high en bloc and R0 resection rates of over 80% [20]. Herein, the en bloc resection rate and R0 resection rate were both 53%, which differs significantly from those reported previously. One reason for this difference may be that the criteria for ESD interventions in our study were different from those used in previous reports. Many of the duodenal ESD cases at our institute are referred from other facilities for unsuccessful resection with polypectomy or EMR. In our study, 33% of the patients had a history of interventions at the same site, which increases the difficulty of removing the submucosa as a whole. Even if no previous interventions were performed, many cases were those in which EMR/hybrid EMR could not be performed due to the size, location, or shape of the lesions. Therefore, en bloc resection is challenging to achieve for such lesions. According to pathological reports, 30% of the lesions are fragmented due to fibrosis, leading to lower R0 resection rates. This report could not demonstrate the advantage of ESD in terms of high en bloc resection rates and R0 resection rates compared with prior studies; however, this may be the standard for duodenal ESD in U.S. facilities. Despite the fact that high en bloc and R0 resection rates could not be achieved, we still consider ESD to be feasible for SNADET resection, as recurrence rates were similar to those previously reported.

This study had some limitations. First, it had a relatively small sample size. This is reflective of the rarity of SNADETs despite their increasing observation. As the U.S. does not have an upper endoscopic medical checkup, unlike eastern Asian countries, it is more difficult to spot SNADETs, limiting the total number of SNADET cases. Second, being conducted at a single tertiary center by an experienced endoscopist may limit the generalizability of the results to other settings. Third, as discussed previously, compared with previous reports, the en bloc and R0 resection rates were lower, and the recurrence rate was similar. This is likely due to the differences in patient backgrounds compared to other reports from different countries. Herein, fibrotic lesions due to previous interventions made complete resection more challenging. Finally, the follow—up period differed between each case, due to the study design.

In the future clinical investigations should be performed with large sample sizes and in multiple medical centers in the U.S. to broaden our understanding of this treatment modality. Follow—up studies over few years to assess any recurrence patterns may be necessary to assess the feasibility of the treatment. Additionally, comparing ESD data with EMR outcomes may be appropriate when more cases with similar patient backgrounds can be obtained.

## 5. Conclusions

To our knowledge, this is the first study to evaluate the outcomes of ESD for SNADETs in North America. As the complication rate was low, we conclude that ESD may be a safe option for SNADETs in the U.S. Lower resection rates and similar recurrence rates were observed compared with previous reports, which is likely a consequence of our unique patient background. Further comparative studies are necessary to determine the most effective approach for in the North American patient population.

## Figures and Tables

**Table 1:** Patient Characteristics.

		N (N = 30)	%
Age		68 Years (47–77)	
Sex	Male	14	47%
	Female	16	53%
Ethnicity	White	26	87%
	African American	4	13%
History of any type of cancer		14	47%
FAP		6 (2 is MAP)	20%
Lynch syndrome		2	7%
Anticoagulation		5	21%
Previous history of interventions at the same site *		10	33%
History of smoking **		7/15	47%
ASA score			
	II	11	37%
	III	19	63%
Anesthesia			
	General Anesthesia	25	84%
	Propofol	5	16%
Location in duodenum			
	Second portion	21	70%
	Third portion	8	27%
	Fourth portion	1	3%

* Interventions include EMR, ESD, polypectomy that are known; ** Smoking history was collected from 15 patients; FAP: familial adenomatous polyposis, MAP: MUYTH associated polyposis; ASA: American Society of Anesthesiologists.

**Table 2:** Endoscopic Procedures and Outcomes.

				%
Procedure time, min, median (IQR) N = 24			142 (100–183)	
Dissection size mm, median (IQR) N =30			30.5 (18–40)	
Dissection area mm^2^, median (IQR) N = 29			535 (283–942)	
Dissection speed mm^2^/min, median (IQR) N = 23			4.27 (3.24–5.42)	
Hospitalized days, median (min, max)			2 (0–7)	
Closure of resected lesion			27	90%
Injection ml, median (IQR)			38 (30–70)	
Histology	En bloc resection		16	53%
	R0 resection		16	53%
	Lateral margin	+	14	47%
		−	16	53%
	Deep margin	+	12	40%
		−	18	60%
	Diagnosis	Adenoma	30	100%
		Adenocarcinoma	0	0%
		Includes high grade adenoma	4	13%
Complications	Post-ESD bleeding (total)		6	20%
	Perforation		0	0%
	Pneumonia		0	0%
Rehospitalization			2	7%
	Post-ESD bleeding		2	100%
Delayed complication	Stenosis		2	7%

ESD: endoscopic submucosal dissection; IQR: interquartile range.

## Data Availability

Data are contained within the article.
